# Medicinal Potential of Isoflavonoids: Polyphenols That May Cure Diabetes

**DOI:** 10.3390/molecules25235491

**Published:** 2020-11-24

**Authors:** Qamar Uddin Ahmed, Abdul Hasib Mohd Ali, Sayeed Mukhtar, Meshari A. Alsharif, Humaira Parveen, Awis Sukarni Mohmad Sabere, Mohamed Sufian Mohd. Nawi, Alfi Khatib, Mohammad Jamshed Siddiqui, Abdulrashid Umar, Alhassan Muhammad Alhassan

**Affiliations:** 1 Department of Pharmaceutical Chemistry, Kulliyyah of Pharmacy, International Islamic University Malaysia, 25200 Kuantan, Pahang DM, Malaysia; hasib76ali@gmail.com (A.H.M.A); awissabere@iium.edu.my (A.S.M.S.); msufian@iium.edu.my (M.S.M.N.); alfikhatib@iium.edu.my (A.K.); jamshed_siddiqui@iium.edu.my (M.J.S.); 2 Pharmacognosy Research Group, Department of Pharmaceutical Chemistry, Kulliyyah of Pharmacy, International Islamic University Malaysia, 25200 Kuantan, Pahang DM, Malaysia; 3 Department of Chemistry, Faculty of Science, University of Tabuk, Tabuk 71491, Saudi Arabia; me_alsharif@ut.edu.sa (M.A.A.); h.nabi@ut.edu.sa (H.P.); 4 Department of Pharmaceutical and Medicinal Chemistry, Faculty of Pharmaceutical Sciences, Usmanu Danfodiyo University, P M B: 2436 Sokoto, Nigeria; abdulrashid.umar@udusok.edu.ng (A.U.); alhasanmaudus@gmail.com (A.M.A.)

**Keywords:** isoflavonoids, biochanin A, genistein, daidzein, glycitein, formononetin, type 2 diabetes mellitus, diabetes complications, mechanism of action

## Abstract

In recent years, there is emerging evidence that isoflavonoids, either dietary or obtained from traditional medicinal plants, could play an important role as a supplementary drug in the management of type 2 diabetes mellitus (T2DM) due to their reported pronounced biological effects in relation to multiple metabolic factors associated with diabetes. Hence, in this regard, we have comprehensively reviewed the potential biological effects of isoflavonoids, particularly biochanin A, genistein, daidzein, glycitein, and formononetin on metabolic disorders and long-term complications induced by T2DM in order to understand whether they can be future candidates as a safe antidiabetic agent. Based on in-depth in vitro and in vivo studies evaluations, isoflavonoids have been found to activate gene expression through the stimulation of peroxisome proliferator-activated receptors (PPARs) (α, γ), modulate carbohydrate metabolism, regulate hyperglycemia, induce dyslipidemia, lessen insulin resistance, and modify adipocyte differentiation and tissue metabolism. Moreover, these natural compounds have also been found to attenuate oxidative stress through the oxidative signaling process and inflammatory mechanism. Hence, isoflavonoids have been envisioned to be able to prevent and slow down the progression of long-term diabetes complications including cardiovascular disease, nephropathy, neuropathy, and retinopathy. Further thoroughgoing investigations in human clinical studies are strongly recommended to obtain the optimum and specific dose and regimen required for supplementation with isoflavonoids and derivatives in diabetic patients.

## 1. Introduction

Diabetes mellitus (DM) is a metabolic disorder related to reduced sensitivity of tissue receptors toward insulin, abnormal insulin signaling, and deteriorating function of β-cells, which lead to abnormal glucose level, sub-clinical inflammation, and increased oxidative stress. DM develops when insufficient or no insulin level is produced (Type 1 diabetes mellitus) or released insulin in the body does not function properly (Type 2 diabetes mellitus), resulting in hyperglycemia [1]. Due to uncontrolled blood sugar, this pathogenic condition leads to long-term serious complications like micro- and macrovascular complications. Microvascular complications generally include neuropathy (nerve damage), nephropathy (renal disease), and retinopathy (vision disorders) while macrovascular complications are coronary artery disease, peripheral arterial disease, and stroke. Due to aforementioned diabetes related complications, specifically macrovascular complications, the risk of ulcer, gangrene, and amputation is greatly increased and consequently affects the quality of life and increase the risk of mortality [2,3]. Type 2 diabetes mellitus is the most prevalent, which consolidates above 90% of the diabetic population and has also been reported to be significantly related to a family history of diabetes, older age, obesity, and lack of exercise [4].

According to the latest data released from the IDF Diabetes Atlas Ninth Edition (2019), approximately 463 million adults (20–79 years) were living with diabetes and by 2045, this number is expected to increase beyond 700 million. The proportion of people with type 2 diabetes has been increasing at an alarming rate in most countries as 79% of adults with diabetes were found to be living in low- and middle-income countries. Moreover, diabetes caused 4.2 million deaths across the world and caused at least USD $760 billion dollars in health expenditure in 2019: 10% of total spending on adults. It has also been projected that 374 million people are at increased risk of developing type 2 diabetes worldwide [5] Diabetes prevalence has been rising more rapidly in low- and middle-income countries than in high-income countries. Due to high worldwide diabetes incidences, the WHO has strongly recommended the implementation of cost and time effective measures be taken immediately against diabetes mellitus treatment to reduce the high morbidity and mortality rate worldwide [6].

There are many drugs available in the management of diabetes mellitus including insulin, α-glucosidase inhibitors (acarbose, miglitol, voglibose), amylin analogs (pramlintide), dipeptidyl peptidase-4 inhibitors (alogliptan, linagliptan, saxagliptin, sitagliptin), incretin mimetics (albiglutide, dulaglutide, exenatide, liraglutide, lixisenatide), meglitinides (nateglinide, repaglinide), non-sulfonylureas (metformin), sulfonylureas (chlorpropamide, glimepiride, glipizide, glyburide, tolazamide, tolbutamide), SGLT-2 inhibitors (canagliflozin, dapagliflozin, empagliflozin), and thiazolidinediones (rosiglitazone, pioglitazone). Though several different types of antidiabetic agents are currently available, the side effects that are generally associated with oral anti-diabetic agents pose grave problems and challenges to tackle this disease efficaciously. Thus, extensive research for alternative but effective and safer therapeutic agents in ameliorating diabetic syndrome has become a critical field in the latest investigations [1,7]. Recently, aldose reductase inhibitors (alrestatin, benurestat, epalrestat, fidarestat, imirestat, lidorestat, minalrestat, ponalrestat, ranirestat, risarestat, sorbinil, tolrestat, zenarestat, and zopolrestat) have been claimed to be an appropriate strategy treatment of diabetic complications [8]. An accumulation of sorbitol has been reported to cause chronic impediments such as peripheral neuropathy, retinopathy, and cataracts. Additionally, aldose reductase has been found to catalyze the reduction of glucose to sorbitol in the polyol pathway, thereby it has been claimed to be associated with the development of diabetic nephropathy [9]. Hence, aldose reductase inhibitors can be seen as a potential avenue in the development of new pharmacological therapy in diabetes mellitus. Similarly, there has been a growing interest in antidiabetic agents from natural products, particularly those derived from traditional medicinal plants [10]. Traditional medicinal plants have always been a rich source of lead compounds and many of the currently available key drugs have been derived either directly or indirectly from them. Hence, the search for more effective and safer therapeutic agents in eradicating diabetic syndromes has continued to be an important area of investigation worldwide [1,7]. In this regard, isoflavonoids could be one of the candidates to effectively tackle the aforementioned problems associated with DM. This review will provide preclinical evidence and elucidate the extent of the pharmacological activities of isoflavonoids, which could prove to be a potential clinical drug in the management of this detrimental disease.

Isoflavonoids are plant based secondary metabolites abundant in plant species that predominantly belong to Papilonoidae, a subfamily of Leguminosae [11]. They are mainly derived via the classical phenylpropanoid pathway. Isoflavonoids are based on a 3-phenylchroman skeleton that is biogenetically derived from a 2-phenylchroman skeleton of flavonoids. Isoflavone aglycone possesses two aromatic rings that are joined together through a heterocyclic pyrane ring [12]. It has been reported that the allocation of the number and position of hydroxyl groups attached to the two aromatic rings influence the biological activity of isoflavonoids [13]. Examples of most studied isoflavones that have been included in this review include biochanin A, genistein, daidzein, glycitein and formononetin (Table 1, Supplementary Materials).

### 1.1. Purpose of the Review

The chief aim of this review was to provide a comprehensive review of the literature regarding the isoflavonoids as well as to establish a critical review of the current evidence in relation to the anti-diabetic potential of plant based isoflavonoids. Hence, this review provides an analysis of the therapeutic potential of isoflavonoids for the slowdown in the progression of DM and possible mechanism of actions. The development of alternatives for insulin secretion control in vitro or in vivo represents an important aspect to be thoroughly explored. In this regard, natural products including isoflavonoids have been progressively explored with this aim. In particular, isoflavonoids, mainly biochanin A, genistein, daidzein, glycitein, and formononetin, have been suggested as potential candidates to act as insulin secretagogue. The information pertaining to antidiabetic potential of the aforementioned isoflavonoids were obtained from different databases and platforms that mainly included PubMed, Scopus, ISI Web of Knowledge, SciELO, Google Scholar, LILACS, Wiley Online Library, Springer Link, Web of Science, and Science Direct. Moreover, abstract proceedings, published books as well as non-indexed and non-impact journals were also taken into deep consideration to gather key information as well as to further ensure that no well documented key information with regard to the antidiabetic potential of the aforementioned isoflavonoids was left out. In this respect, a total of 134 articles from 1992 to 2020 focused on antidiabetic activity was identified. After thorough study and investigations of all searched articles, 116 manuscripts were finally selected based on the exclusion and inclusion criteria to accomplish this review. More precisely, for this review, the inclusion criteria thoroughly consisted of research articles published in indexed as well as non-indexed journals, year of publication, and in vitro and in vivo antidiabetic investigations (antihyperglycemic and hypoglycemic). Exclusion criteria comprised the repetition of similar effect with the same compound, insignificant results with poor statistical analysis, poorly written articles, article published twice in different journals with duplicate content, weakly orchestrated research work as well as research manuscripts that did not fulfil the aforementioned inclusion criteria.

### 1.2. Dietary Sources and Intake of Isoflavonoids

Soy and its products, and legume seeds (lentils, beans, peas) are one of the richest sources of isoflavones in normal food. Additionally, soybean seeds are an essential source that contain a massive amount of isoflavonoids in the human diet. However, the main sources of isoflavones are from legumes, particularly the Fabaceae family in soy and red clover [14]. Furthermore, isoflavones can also be found in trace quantities in some fruits such as strawberries and apples [15].

It has been reported that the daily intake of isoflavones in Asia varies among regions and subpopulation in the same country, ranging from 15 to 50 mg. This is also supported by results of other research studies in which it was stated that the daily isoflavone intake among Southeast Asians was between 15 and 47 mg [16,17,18]. However, in Western countries, people have been found to consume far less isoflavones, between 0.15 mg and 1.7 mg per day [19,20,21].

As far as their composition, either in soy or red clover is concerned, they also totally differ. For instance, daidzein, genistein, and glycitein are predominantly found as major isoflavones in soy whilst, formononetin and biochanin A are found as major isoflavones mainly in red clover. However, daidzein and genistein exist as minor constituents [14].

### 1.3. Biological Importance of Isoflavonoids

Isoflavonoids have become an interesting phytoconstituent toward their production as dietary intake supplements for the purpose of health benefits due to their various favorable biological effects on human health. Several studies have reported that isoflavonoids may contribute multiple additional health benefits by reducing cardiovascular risk, osteoporosis, and decreasing the intensity of bone resorption [22]. They have also been reported to exert multiple in vitro and in vivo pharmacological effects including antioxidant, anticancer, antimutagenic, antiproliferative, anti-diabetic, and estrogenic effects [23,24,25,26,27,28]. Moreover, Molteni et al. [29] reported that isoflavonoids had a structure resemblance with the β-estradiol and possessed affinity toward estrogen receptors. This further supports the association of isoflavonoids with anticancer effects against breast cancer and post-menopausal abnormality [30].

Jang et al. [23] mentioned that isoflavonoid aglycones seemed to have greater antidiabetic activity in soy fermentation, which led to opening a new window of knowledge among researchers to explore and reveal the potential of isoflavonoids that show various biological effects in human health. This was supported by Kawakami et al. [31], who claimed that an isoflavone aglycone-rich diet reduced liver and total cholesterol levels as well as triglyceride levels in rats fed with cholesterol. This indicates that increased isoflavonoid aglycone content may be effective in ameliorating metabolic disorders [32].

Epidemiological and clinical trials have proposed a positive role for isoflavonoids in human health and nutrition [33]. Numerous epidemiological studies and growing evidence have been linked to the attribution of isoflavonoids in dietary intake, related to its wide spectrum of health benefits such as immunomodulation, risk reduction of chronic diseases including cardiovascular diseases, diabetes, cancer, osteoporosis and obesity [25] as well as diminished menopausal symptoms [26,27].

## 2. Discussion

### 2.1. Metabolism and Bioavailability of Isoflavonoids

In general, most isoflavonoids exist in plants as glycosides. Setchell et al. [34] suggested that hydrolysis of the sugar moiety is important for absorption of isoflavonoids. However, Zheng et al. [35] and Zubik and Meydani [36] reported that there was no difference between the absorption of aglycone and glycosides. Although some researchers found that the aglycone moiety was more systemically absorbed in the body [37,38], this absorption process did not seem to be related to the food chain and processing or isoflavone dietary source [39].

**Figure 1:**
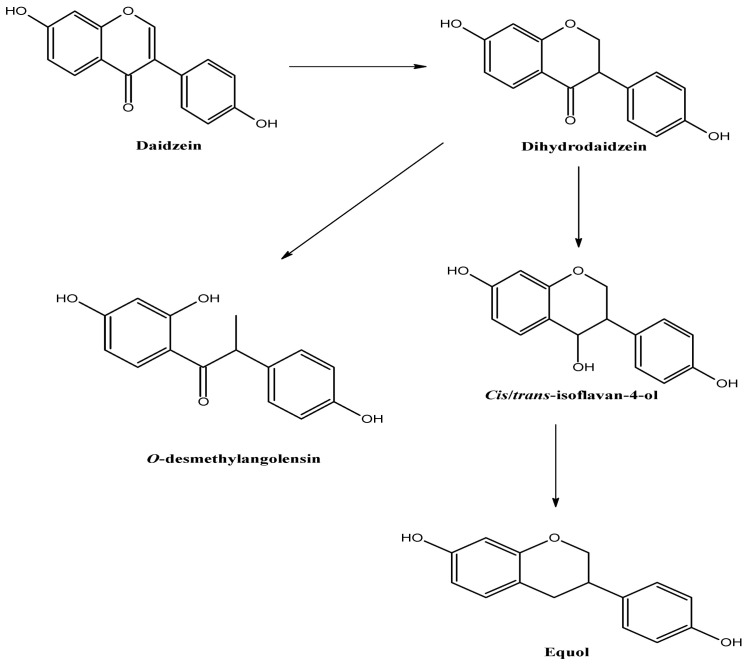
Metabolism of the daidzein to equol and *O*-desmethylangolensin. Adapted from Heinonen et al. [42].

β-glucosidase catalyzes the hydrolysis of the glycosidic bonds to terminal non-reducing residues in β-d-glucosides and oligosaccharides with the release of glucose. Day et al. [40] reported that the aglycone portion upon the hydrolysis of glycoside of isoflavonoids is further metabolized in the gut microflora environment. This explains the metabolism process of formononetin and biochanin A to produce demethylated metabolites to form daidzein and genistein, respectively. Moreover, daidzein is further metabolized by colonic bacteria to form equol or *O*-desmethylangolensin (ODMA) via a pathway that involves the formation of the intermediate dihydrodaidzein (Figure 1). Hwang et al. [41] evaluated the interaction of all aforementioned metabolites of different isoflavonoids against estrogen receptors and found that ODMA had the weakest affinity among all metabolites.

### 2.2. Mode of Action as an Antidiabetic Agent

Several studies have been carried out to explore the potential role of isoflavonoids in the treatment of diabetes mellitus. Results from in vitro and in vivo studies have proven the hypoglycemic effect of isoflavonoids. It has also been testified that isoflavonoids can act as an insulin secretagogue that induces insulin secretion to ameliorate diabetic complications. Furthermore, it has been claimed that isoflavonoids improve insulin resistance and stimulate glucose uptake in peripheral tissues that control the rate limiting enzymes involved in the carbohydrate metabolism pathway. Hence, isoflavonoids are nowadays indicated as potential significant natural compounds to enrich the recent therapy options against the manifestation of diabetes mellitus [43,44].

#### 2.2.1. Peroxisome Proliferator-Activated Receptors α and γ

Isoflavonoids have been reported to activate the ligand-dependent transcription factors known as peroxisome proliferator-activated receptors (PPARs), which play an important role in gene expression in diabetes mellitus [45]. Moreover, PPARs have been found to be highly associated with the gene expressions involved in adipogenesis, lipid metabolism, inflammation, and the maintenance of metabolic homeostasis [46]. Additionally, PPARγ has been reported to be the central regulator of adipocyte differentiation, important in regulating lipid metabolism and glucose homeostasis [47,48,49] and Wang et al. [46] suggested that agonists of PPARγ could be therapeutically considered to counter hyperglycemia elicited due to type 2 diabetes. An oral antidiabetic agent, thiazolidinedione, which acts as an agonist of PPARγ, has been reported to exert serious side effects, making the discovery of novel ligands highly relevant that can lead to the discovery of potentially safe antidiabetic agents. In this regard, Shen et al. [50] stated that compounds that target PPARα and PPARγ could potentially be used to regulate dyslipidemia and restore glycemic balance, respectively. Due to the fact that many diabetic patients suffer from atherogenic lipid abnormalities and insulin resistance, ligands that may stimulate both PPARα and PPARγ could be efficaciously used in obese patients with type 2 diabetes.

In this regard, Mezei et al. [51] reported that isoflavonoids exhibit antidiabetic and hypolipidemic effects through the PPAR pathway in obese Zucker rats. Shen et al. [50] also found that isoflavonoids viz. formononetin and calycosin isolated from *Astragalus membranaceus* (Fisch.) Bunge exerted significant activation of PPARα and PPARγ. Formononetin showed higher activity, which was comparable to what had been observed for some synthetic dual PPAR-activating compounds, further supporting its antidiabetic potential to be used in regulating lipid metabolism. Furthermore, this study was in line with the study carried out by Qiu et al. [52], who reported that red clover extract containing biochanin A and formononetin ameliorated dyslipidemia in streptozotocin-induced diabetic C57BL/6 mice by activating hepatic PPARα as well as significantly downregulating hepatic APOC3 expression. The therapeutic potential of the active fraction containing isoflavonoids, namely calycosin, formononetin, ononin, and calycosin-7-*O*-β-d-glucoside from the same plant was validated. Continuous administration of the fraction considerably improved the glycemic control, reduced the levels of serum triglyceride, and also alleviated insulin resistance and glucose intolerance in db/db obese mice. These effects were the result of its anti-inflammatory activity [53]. Wang et al. [46] also attributed the hypoglycemic effect of biochanin A and genistein to stimulation of the PPARα and PPARγ receptors. Additionally, biochanin A was also found to induce an in vitro adipocyte differentiation stimulation through PPARγ as measured by gene activity [54]. Genistein and daidzein have also been reported to activate PPARα and PPARγ as well as modulate adipocyte differentiation in vitro [45]. Cho et al. [55] reported that equol, which is a metabolite of daidzein, enhances adipocyte differentiation and PPARγ transcriptional activity. This study was further supported by Cheong et al. [56], who reported that equol exerted an in vitro antidiabetic effect in cultured L6 myocytes by promoting glucose uptake, AMPK phosphorylation, and GLUT4 translocation detected by western blotting analyses in L6 myotubes under a condition of insulin absence. In the same study, an in vivo antidiabetic effect of equol in obese-diabetic model ob/ob mice was also investigated. Equol (0.05% in diet) suppressed the rise in serum glucose, cholesterol, triglyceride, and lipid peroxide concentrations and the hepatic triglyceride level compared with those in the control group. Furthermore, equol treatment was found to suppress the rise in fasting blood glucose level and improve the impaired glucose tolerance in ob/ob mice. Moreover, equol treatment was also found to improve the expression of hepatic gluconeogenesis- and lipogenesis-related genes in terms of glucose and lipid metabolism. The hypoglycemic effect of equol was found to be related to the GLUT4 translocation increment in the plasma membrane through AMPK activation. In addition, equol was also found to suppress the fasting blood glucose level and gene expression of hepatic enzymes related to glucose metabolism. These results strongly support equol in its role as an antidiabetic agent.

Since the insulin sensitizing effect of an anti-diabetic drug, namely thiazolidinedione, is mediated through PPARγ, an extensive search has been done using 3T3-L1 cells in vitro. Cho et al. [55] claimed that daidzein enhanced insulin-stimulated glucose uptake in adipocytes by increasing the expression of GLUT4 and IRS-1 via the stimulation of PPARγ. This was further supported by Kwon et al. [57], who reported that daidzein had better insulin-sensitizing actions by activating PPARγ in 3T3-L1 adipocytes. Carrara et al. [58] also previously mentioned the activity of daidzein and glycitein in the activation of PPAR transcriptional activity in human promonocytic U-937 cells. These results confirm that plant isoflavonoids activate gene expression through the stimulation of PPARα and PPARγ, and dietary isoflavonoids regulate adipogenesis, lipid metabolism, inflammation, and the maintenance of metabolic homeostasis.

#### 2.2.2. Antihyperglycemic Effect of Isoflavonoids with Regard to Carbohydrate Metabolism, Insulin Sensitivity and Insulin Resistance, and Preservation of β-Cell Function

It has been confirmed that abnormal digestion and malabsorption of dietary carbohydrate are mainly responsible for reduced glycogen storage, increased synthesis of glucose and overproduction of hepatic glucose, β-cell impairment, insulin resistance of peripheral tissue, and defect in insulin signaling pathways, which greatly contribute to the manifestation of diabetes mellitus [59]. The hypoglycemic effect of isoflavonoids has also been reported to be mainly associated with the reduced intestinal absorption of dietary carbohydrates, modulation of the enzymes involved in glucose metabolism, improvement of β-cell functions and insulin action, stimulation of insulin secretion and the antioxidative as well as anti-inflammatory attributes of these compounds. One of the most well-known properties of isoflavonoids on carbohydrate metabolism is the intestinal inhibition of *α*-glucosidase on the hydrolysis of carbohydrates. In a study done by Lee and Lee [60], they claimed that genistein is a potent inhibitor of *α*-glucosidase obtained from fermentation broths of a *Streptomyces* sp. Choi et al. [61] also reported that biochanin A, genistein, formononetin, and daidzein exhibited a potent in vitro inhibition on yeast α-glucosidase in a dose dependent manner.

Non-enzymatic glycosylation of proteins is considered as the major cause of diabetic complications. It seems that protein glycosylation can be inhibited effectively by antioxidants. Hence, in one of the studies, biochanin A was selected to determine its antioxidant effects on in vitro insulin, hemoglobin, and albumin glycosylation. The optimal glucose concentration and incubation time were obtained for each protein. Then, the inhibition percentage of protein glycosylation was measured in the presence of three different concentrations (0.5, 5, and 10 µg/mL) of biochanin A by a colorimetric method. The results demonstrated that biochanin A strongly inhibits insulin and hemoglobin glycosylation by inhibiting their glycosylation 100% and 60%, respectively. Biochanin A also inhibited glycosylation of albumin 100%. Therefore, it seems probable that plants containing isoflavonoids may have preventive effects in diabetic complications [62]. In an another study, biochanin A showed an antihyperglycemic effect in streptozocin-diabetic rats [63]. This statement was correlated and agreed with another study carried out by Azizi et al. [64], who mentioned that biochanin A showed a beneficial effect on the reduction of fasting blood glucose (FBG), body weight, reduction of glycosylated hemoglobin (HbA1c), lipid profile, and serum enzymes in streptozocin-induced diabetic rats. Furthermore, several studies have also highlighted the potential antidiabetic effect of isoflavonoids, in which they have been reported to be able to regulate postprandial glycemia and inhibit development of glucose intolerance by facilitating insulin response as well as inducing insulin secretion. Vedavanam et al. [65] mentioned that soya extract containing genistein and daidzein, in particular, acts as an inhibitor of intestinal glucose-uptake and as a preventive agent for glucose-induced lipid peroxidation in vitro. Kwon et al. [57] reported that daidzein is capable of generating insulinotropic action by stimulating glucagon-like peptide 1 secretion in enteroendocrine NCI-H716 cells. This was, however, not in line with the work carried out by Higashi and Ogawara [66], who stated that daidzein inhibits insulin like growth factor 1 mediated signaling in cell cycle progression of Swiss 3T3 cells. This statement, however, was not consistent with Kwon et al. [67], who stated that daidzein could enhance insulin-stimulated glucose uptake in 3T3-L1 adipocytes. Meanwhile, this statement is supported through an in vivo study done by Oh et al. [68], who claimed that daidzein decreased blood glucose levels in streptozotocin-induced diabetic rats. Later, Cheong et al. [69] reported that daidzein improved glucose homeostasis in type 2 diabetic mice model, thereby suggesting its role as an antidiabetic agent. Interestingly, a systematic study done by Getek et al. [70] further confirmed that leguminous plants particularly containing genistein and daidzein exhibited in vitro and in vivo antidiabetic activities. Hence, they emphasized that such leguminous plants should be employed in the promotion of a healthy lifestyle as a form of functional food and should constitute a permanent dietary element in a balanced diet, especially with type 2 diabetic patients. More recently, Lee et al. [71] isolated formononetin from *A. membranaceus* and examined on glucose-stimulated insulin secretion (GSIS) from pancreatic β-cell. It was found to stimulate insulin secretion in INS-1 cells without inducing cytotoxicity. A further evaluation demonstrated that formononetin enhanced the phosphorylation of total insulin receptor substrate-2 (IRS-2), phosphatidylinositol 3-kinase (PI3K), and Akt, and activated pancreatic and duodenal homeobox-1 (PDX-1) and PPAR-γ, which are associated with β-cell function and insulin secretion. The data suggest that formononetin has the potential to improve insulin secretion in β-cell, representing the first step toward the development of potent antidiabetic drugs.

Many studies have been done to determine whether isoflavonoids may play any role in reversing the insulin resistant effect in diabetes. More recently, an in vitro study was done by Li et al. [72] to determine the anti-diabetic effect of genistien, biochanin A, and formononetin based derivatives using an insulin-resistant (IR) HepG2 cell model. The results indicated that sulfated, esterified and prenylated derivatives of biochanin A, chromium complex of biochanin A, and genistein exhibited significant glucose consumption-enhancing effects in IR-HepG2 cells. In addition, the combinations of these derivatives displayed better anti-diabetic activity than the individual compounds. This study provides useful clues for the further design and discovery of safe anti-diabetic agents. In this regard, Wei et al. [73] further studied the hypoglycemic activity of derivatives of isoflavones from *Cicer arietinum* L (chickpea), a food and medicine used by the people of Xinjiang that has a beneficial hypoglycemic effect. To better utilize this national resource and develop hypoglycemic agents from components of the chickpea, a series of new derivatives of isoflavone compounds from the chickpea was synthesized. An insulin-resistant (IR) HepG2 cell model was used to screen the hypoglycemic activities of these derivatives of isoflavones. Several combinations of these derivatives of isoflavones displayed higher hypoglycemic activity than any single compound, and they had similar hypoglycemic activity to that of the positive control group (*p* > 0.05). These findings demonstrate the characteristics of derivatives of isoflavones in herbal medicine. This evidence was suggested to provide new innovative ideas for the development of hypoglycemic drugs.

Similarly, formononetin has further been reported to show the best effect in an IR HepG2 cell model [72] and reduce caspase-3 levels in the INS-1 cell line [74]. Additionally, genistein directly inhibited GLUT4-mediated glucose uptake in 3T3-L1 adipocytes [75] and stimulated glucose uptake in L6 myotubes [76]. This study showed correlation with the study that had earlier been done by Smith et al. [77] who mentioned that genistein inhibited insulin-stimulated glucose transport and reduced immunocytochemical labeling of GLUT4 carboxyl-terminus. Genistein also showed an inhibitory mechanism on insulin-stimulated glucose uptake in MC3T3-G2/PA6 adipose cells, which showed a good indicator of glucose homeostasis [78].

Several in vitro and in vivo studies have been reported on genistein acting as an insulin secretagogue. Jonas et al. [79] reported that genistein stimulates an in vitro insulin release in normal mouse islets. Liu et al. [80] claimed that genistein increases glucose-stimulated insulin secretion in both insulin secreting cell line (INS-1 and MIN6) and mouse pancreatic islets. This study was in line with the previous study done by Ohno et al. [81] who reported that genistein improved and increased insulin secretion (in a dose-dependent manner up to 20 μg/mL) by increasing the calcium concentration into an intracellular medium that characterizes the primary signal in the regulation of insulin secretion and cyclic adenosine 3′5′-monophosphate (cAMP), an important molecule that acts as a type of cellular secondary messenger. When activated, the cAMP manifests a regulatory effect in multiple peripheral tissues, enhances insulin sensitivity, stimulates glucose uptake, and promotes gene expression. It might also generate ATP into cells and activate CaMK II. CaMKII participates in GSIS in several steps of this process such as the modulation of cytoplasmic content of ATP and the synthesis of insulin granules [82]. Furthermore, Fu and Liu [83] demonstrated that genistein augments the insulin secretory function of pancreatic β-cells in clonal insulin secreting (INS-1E) cells. Neye and Verspohl [84] mentioned that genistein inhibited tyrosine kinase in the INS-1 cell line, which was in line with the study done by Lee et al. [76], who clarified that genistein amplified insulin secretion in INS-1 cells.

There are many in vivo and in vitro research studies that display, at physiologically-relevant concentrations (<10 µM), a direct effect of genistein on β-cells. The effects seem to involve cAMP/PKA signaling and there are some studies that suggest an effect on the epigenetic regulation of gene expression [85]. Past research has reported that genistein differentially inhibits the post-receptor effects of insulin in rat adipocytes without inhibiting the insulin receptor kinase [86]. Moreover, genistein was also reported to induce adipogenesis, but inhibited leptin production in human synovial fibroblasts [87]. This study was also found to correlate with the work carried out by Szkudelski et al. [88], who mentioned that genistein restricted leptin secretion from rat adipocytes. Additionally, genistein was also found to reverse the antilipolytic action of insulin in isolated rat adipocytes [89].

Apart from that, genistein plays an important role in regulating the glucose level in blood as well as induces insulinotropic action viz. stimulating or affecting the production and activity of insulin (an insulinotropic hormone). In fact, genistein exhibited significant glucose consumption-augmenting effects in IR-HepG2 cells [72]. This study was also in line with Chen et al. [90], who mentioned that genistein modified basal glucose uptake in HepG2 cells. In addition, Kwon et al. [57] demonstrated that genistein generated an in vitro insulinotropic action by stimulating glucagon-like peptide-1 secretion in enteroendocrine NCI-H716 cells.

Several in vivo studies carried out on determining the genistein’s anti-diabetic potential revealed that it exerts its antidiabetic action by regulating basal glucose level, insulin release as well as protecting the functions of β-cell located in the islets of Langerhans. Fu et al. [91] investigated the effect of genistein on β-cell proliferation and cellular signaling related to this effect and further evaluated its antidiabetic potential in insulin-deficient diabetic mice. Genistein induced both INS1 and human islet β-cell proliferation after 24 h of incubation, with 5 mum genistein inducing a maximal 27% increase. The effect of genistein on β-cell proliferation was neither dependent on estrogen receptors nor shared by 17 β-estradiol or a host of structurally related flavonoid compounds. Pharmacological or molecular intervention of protein kinase A (PKA) or ERK1/2 completely abolished genistein-stimulated β-cell proliferation, suggesting that both molecules are essential for genistein action. Consistent with its effect on cell proliferation, genistein induced cAMP/PKA signaling and subsequent phosphorylation of ERK1/2 in both INS1 cells and human islets. Furthermore, genistein induced protein expression of cyclin D1, a major cell-cycle regulator essential for β-cell growth. Dietary intake of genistein significantly improved hyperglycemia, glucose tolerance, and blood insulin levels in streptozotocin-induced diabetic mice, concomitant with improved islet β-cell proliferation, survival, and mass. These results support that genistein might be a natural antidiabetic agent by directly modulating pancreatic β-cell function via activation of the cAMP/PKA-dependent ERK1/2 signaling pathway.

It is known that peripheral insulin resistance is common during obesity and aging in mice and people. The progression to T2DM is largely due to loss of β-cell mass and function through apoptosis [92]. Fu et al. [93] further evaluated whether genistein could prevent β-cell loss in T2DM mice. Hence, the effect of dietary supplementation of genistein on glycemic control and β-cell mass and function was determined. Dietary intake of genistein (250 mg/kg diet) improved hyperglycemia, glucose tolerance, and blood insulin level in obese diabetic mice, whereas it did not affect body weight gain, food intake, fat deposit, plasma lipid profile, and peripheral insulin sensitivity. Genistein was found to increase the number of insulin-positive β-cells in islets, promoted islet β-cell survival, and preserved islet mass. In conclusion, it was suggested that dietary intake of genistein could prevent T2DM via a direct protective action on β-cell without alteration of periphery insulin sensitivity.

Wang et al. [94] further evaluated the antidiabetic activity of genistein by observing insulin sensitivity in mice. Results of this study showed that genistein weakened glucose tolerance and reduced insulin sensitivity by inhibiting the insulin-induced phosphorylation of insulin receptor substrate-1 (IRS1) at tyrosine residues, leading to the inhibition of insulin-mediated GLUT4 translocation in adipocytes. Mac-CM, an inflammatory stimulus, induced glucose intolerance accompanied by impaired insulin sensitivity; genistein reversed these changes by restoring the disturbed IRS1 function, leading to an improvement in GLUT4 translocation. In addition, genistein increased AMPK activity under both normal and inflammatory conditions; this was shown to contribute to the anti-inflammatory effect of genistein, which led to an improvement in insulin signaling and the amelioration of insulin resistance. Hence, it was concluded that genistein showed the opposite effects on insulin sensitivity under normal and inflammatory conditions in adipose tissue and this action was derived from its negative or positive regulation of IRS1 function. Its upregulation of AMPK activity contributes to the inhibition of inflammation implicated in insulin resistance. This finding was found to be actually correlated with a study that was done earlier using non-diabetic mice in which genistein boosted insulin level and altered hepatic gluconeogenic and lipogenic enzyme activities [95]. Liu et al. [80] explained that genistein stimulates insulin secretion in a dose of 0.01–5 μM, thereby stimulates intracellular cAMP accumulation, which consequently activates protein kinase A (PKA) via protein tyrosine kinase (PTK) independent and estrogen receptor independent. Furthermore, genistein was also found to promote the insulin signaling pathway in the cerebral cortex of aged female rats [96].

Oh et al. [68] evaluated the hypoglycemic action of semipurified fractions obtained from hot-water extract of the submerged-culture broth of *Agaricus blazei* Murill in streptozotocin (60 mg/kg, intraperitoneal)-induced diabetic male Sprague-Dawley rats, relative to the diabetes drug metformin. Prior to investigation, the hot-water extract was treated with ethanol to remove β-glucans and glycoproteins, freeze-dried, and fractionated into hexane, chloroform, ethyl acetate (EA), and butanol fractions. In a 14 day-treatment study, the EA fraction (200 mg/kg body weight) was found to reduce (*p* < 0.05) the blood glucose level in the oral glucose tolerance test, relative to the other fractions and control. It was also found to elevate plasma insulin and glucose transport-4 proteins as well as reduce the levels of triglyceride and cholesterol in plasma, the activity of glutamate-oxaloacetate transaminase and glutamate-pyruvate transaminase in blood, and the content of thiobarbituric acid reactive substance in the liver and kidney. The hypoglycemic efficacy of the EA (400 mg/kg body weight) was found to be similar to that of metformin (500 mg/kg body weight). Upon phytoconstituent characterization of the EA fraction, it was found to contain a substantial number of isoflavonoids including genistein, genistin, daidzein, and daidzin, which were suggested to have contributed to the EA fraction’s hypoglycemic action. These results indicate that the hot-water extract of the submerged-culture broth of *A. blazei* contains an EA fraction with potent hypoglycemic action, which could be useful in the treatment of diabetes mellitus.

Rauter et al. [97] also evaluated the antidiabetic effect of genistein isolated from *Genista tenera* on streptozotocin (STZ)-induced diabetic Wistar rats. The genistein and genistein 7-O-glucoside, along with another six flavonoids (viz. apigenin, chrysoeriol, apigenin 7-O-glucoside, luteolin 7-O-glucoside, rutin, and luteolin 7,3′-di-O-glucoside) were administered intraperitoneally for seven days (4 mg/kg body weight/day). The protective effect of these compounds over the liver and kidneys of STZ-diabetic models was also evaluated by the determination of seric AST, ALT, and urea levels. After seven days of treatment, apigenin, chrysoeriol, and genistein significantly lowered the blood glucose levels of diabetic animals. This effect was found to be more pronounced (*p* < 0.01) in the oral glucose tolerance test. Glucose tolerance was also significantly improved in the rutin (*p* < 0.01) and in the genistein 7-O-glucoside (*p* < 0.05) treated groups. In addition, almost all the tested flavonoids effectively protected the liver and kidneys against STZ-induced damage in rats. Moreover, Jesus et al. [98] synthesized 8-β-d-glucopyranosylgenistein (the major component of *Genista tenera*) and evaluated its therapeutical impact in the treatment of STZ-induced diabetic rats. It was found to produce normalization of fasting hyperglycemia and amelioration of excessive postprandial glucose excursions and increasing β-cell sensitivity, insulin secretion, and circulating insulin within seven days at a dose of 4 (mg/kg body weight/day). These results support it as a potential antidiabetic agent. Genistein treatment was also able to endorse estrogen-like effects in ovariectomized (OVX) Sprague-Dawley rats [99]. This study was found to be correlated with a study done by Nogowski et al. [100], who reported that genistein influenced insulin receptors and insulin secretion in OVX rats. A similar result was observed in a more extensive study done by Maćkowiak et al. [101] in which genistein was shown to influence the effect of insulin receptors in perfused liver of ovariectomized rats. Ae Park et al. [102] reported that genistein was able to modulate hepatic glucose and lipid regulating enzyme activities in C57BL/KsJ-db/db mice. Meanwhile, Weigt et al. [103] added that genistein showed a molecular effect of ER α- and β-selective agonists on the regulation of energy homeostasis in obese female Wistar rats. All of these in vivo studies suggest that genistein is capable of regulating the homeostasis of blood glucose level, lipid peroxidation, and insulin resistance as well as β islet pancreatic modulation. This was further supported by the study by Arunkumar and Anuradha [104], who mentioned that genistein improved insulin action in the muscle by targeting AMPK in a high fructose diet fed-mice model of insulin resistance.

Meanwhile, daidzein has also been reported to show antidiabetic potential through multiple in vitro as well as in vivo antidiabetic studies. It has been reported to demonstrate its antidiabetic effects in terms of its regulation in carbohydrate metabolism, insulin modulation, and insulin resistance as well as adipocyte homeostasis and induced insulin secretion. Park et al. [105] reported that daidzein inhibited carbohydrate digestive enzymes in vitro. Daidzein showed prominent inhibitory effects against α-glucosidase and α-amylase. The half maximal inhibitory concentration (IC_50_) values of daidzein against α-glucosidase and α-amylase were 0.048 and 0.301 mmol, respectively, which suggested that daidzein was more effective than acarbose. An in vitro anti-diabetic activity of diadzein was further explained by promoting glucose uptake through glucose transporter 4 translocation to the plasma membrane in L6 myocytes [69]. Seo et al. [106] evaluated the anti-adipogenic effect of a metabolite of daidzein known as 6,7,4’-trihydroxyisoflavone. It was found to suppress adipogenesis in 3T3-L1 preadipocytes via ATP-competitive inhibition of P13K. Therefore, it may have potential for development into novel treatment strategies for chronic obesity and diabetes. Choi et al. [95] reported that the genistein and daidzein supplements increased the insulin/glucagon ratio and C-peptide level with preservation of insulin staining β-cells of the pancreas in the NOD mice. In the liver, genistein and daidzein supplements resulted in lowering glucose-6-phosphatase (G6Pase) and phosphoenolpyruvate carboxykinase (PEPCK) activities, while increasing two lipogenic enzymes activities, malic enzyme and glucose-6-phosphate dehydrogenase (G6PD) compared to the control group. Moreover, genistein and daidzein supplementation lowered the activities of fatty acid β-oxidation and carnitine palmitoyltransferase (CPT) in these mice. Genistein and daidzein also improved plasma triglyceride and free fatty acid (FFA) concentrations compared to the control group. These results suggest that both isoflavonoids play important roles in the regulation of glucose homeostasis in type 1 diabetic mice by downregulating G6Pase, PEPCK, fatty acid β-oxidation, and CPT activities, while upregulating malic enzyme and G6PD activities in the liver with the preservation of pancreatic β-cells. The supplementation of genistein and daidzein are seemingly helpful for preventing insulin-dependent diabetes mellitus (IDDM) onset.

Apart from the above, daidzein has also been claimed to reduce serum insulin and insulin resistance in an animal model of obesity and diabetes [107]. Ae Park et al. [102] reported that daidzein also modulated hepatic glucose and regulate lipid enzyme activities in C57BL/KsJ-db/db mice. This statement was later supported by another study in which it was clearly mentioned that the mixture of daidzein and glycitin demonstrated anti-obese and anti-diabetic effects in C57BL/6J mice fed with a high-fat diet [108]. Park et al. [105] also evaluated daidzein’s in vivo antidiabetic effects. The results indicated that the postprandial blood glucose level was more significantly suppressed in the daidzein-administered group than in the water group of both streptozotocin-induced diabetic and normal mice. These results indicate that daidzein may be a potent antidiabetic agent. In summary, results of the studies clearly acknowledge that isoflavonoids favorably affect several aspects of diabetes-induced metabolic disorders and modulate carbohydrate metabolism, glucose homeostasis, insulin secretion, and insulin resistance as well as β islet pancreatic cell protection. Hence, they could be regarded as potent antidiabetic agents.

#### 2.2.3. Antioxidative Properties of Isoflavonoids in Prevention of Long Term Diabetes Complications

An antioxidant property is one of the most important biological properties exerted by the isoflavonoids. This effect has been suggested to facilitate prevention and slow progression against long-term diabetes complications including retinopathy, nephropathy, and neuropathy. It has been suggested that phytoestrogens that bear more than one hydroxyl group may be a more effective antioxidant against peroxyl radicals. It was also mentioned that antioxidant activity of genistein was due to conjugation of the C2–C3 double bond with 4-oxo atom in the A and C rings [109].

Asgary et al. [62] reported that biochanin A demonstrated inhibitory activity on protein glycosylation in diabetic retinopathy. Vedavanam et al. [65] demonstrated that genistein containing soybean phytochemical extract exhibited antioxidant action. Persaud et al. [110] claimed that genistein inhibited islet tyrosine kinase activity and glucose-, 4-α ketoisocaproic acid (KIC), and sulfonylurea-stimulated insulin release without affecting glucose metabolism. Genistein also inhibited protein serine/threonine kinase activities to a limited extent, but had no effect on Ca^2+^, cyclic AMP- or phorbol myristate acetate (PMA)-induced insulin secretion from electrically permeabilized islets. These results suggest that genistein exerts its inhibitory effect on insulin secretion proximal to Ca^2+^ entry and it was proposed that it acts at the site of the voltage-dependent Ca^2+^ channel that regulates Ca^2+^ influx into β-cells following nutrient- and sulfonylurea-induced depolarization.

Furthermore, Cheng et al. [111] showed that genistein exerted a hypoglycemic effect on a rat experimental model of postmenopausal metabolic syndrome [112]. Genistein also modified liver function and mitigated non-alcoholic fatty liver disease in a rat model of insulin resistance [113]. Interestingly, Lee [114] reported that genistein showed antioxidant enzyme activity and controlled the blood glucose homeostasis and lipid profile in streptozotocin-induced diabetic rats. Meanwhile, a metabolite of daidzein, specifically equol, has been reported to exert a suppressive effect against alloxan-induced oxidative stress in INS-1 pancreatic β-cells [115].

It is suggested that the 5C position located on the A ring of genistein explains the antioxidant activity on β-cell proliferation since equol and 17 β-estradiol lack this hydroxyl group and were unable to stimulate β-cell proliferation. Importantly, when glucose (genistein) replaced the hydroxyl group at position 7C or at the 4C position with a methyl group (biochanin A), there was no difference in β-cell proliferation, suggesting that these structural parts are not important for demonstrating the antioxidants effects of genistein on β-cell proliferation [91]. Moreover, Ateba et al. [116] highlighted the importance of the prenylation of flavonoids to enhance various biological activities compared to the respective non-prenylated flavonoid. In line with this, the natural prenylated isoflavonoid alpinumisoflavone (AIF) has been explored for a number of biological and pharmacological effects (therapeutic potential). Reported data showed that AIF had a multitherapeutic potential with antiosteoporotic, antioxidant and anti-inflammatory, antimicrobial, anticancer, estrogenic and antiestrogenic, antidiabetic, and neuroprotective properties. In a nutshell, study on the biological activity of isoflavonoids is important and the structural specificity needs to be considered since the modification of the chemical structure to various metabolites through the addition of the sugar group or methyl group or prenylation mediates the antioxidant activity that is associated with the antidiabetic activity of isoflavonoids. The rising trends in the prevalence of diabetes complications indicate that recent medical treatments for the management of diabetes are not sufficient and that the practice of supplementary intake treatments including functional foods and their nutraceuticals could augment the effectiveness of diabetes management. Plant isoflavonoids based on in vitro and in vivo studies have been suggested to be as effective and beneficial alternatives for diabetes management and the prevention of its long-term complications.

## 3. Conclusions

Isoflavonoids are naturally derived phytoestrogen compounds that have been reported to exert in vitro and in vivo antidiabetic effects through multiple research studies with different mechanisms of actions. Biochanin A, genistein, daidzein, glycitein, and formononetin are the main promising isoflavonoids that have been thoroughly investigated for their antidiabetic potential. Among these isoflavonoids, genistein may represent a promising candidate for an alternative approach to prevent and treat diabetes. Biochanin A also opened up new horizons as it has been demonstrated to exert antidiabetic action by inhibiting aldose reductase as well as through PPAR α- and γ-modulation pathways that regulate glycemic balance. Structure activity relationship studies suggest that isoflavones that possess more than one hydroxyl groups attached to the aromatic rings may be a more effective antioxidant against peroxyl radicals. However, studies on these aspects of isoflavonoids are not sufficient and need to be re-evaluated using more appropriate methods and methodology. Hence, future studies are still warranted to understand the exact structural specificity of isoflavonoids that may bring specific antidiabetic activity toward a specific mechanism in treating diabetes mellitus. Furthermore, further investigations in human clinical studies are highly needed to obtain the optimum and specific dose and regimen required for supplementation with isoflavonoids in diabetic patients.

## Figures and Tables

**Table 1:** Structure of isoflavones: (1) biochanin A, (2) genistein, (3) daidzein, (4) glycitein, and (5) formononetin.

No	Isoflavonoids	Biological Structure
1	Biochanin A	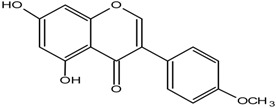
2	Genistein	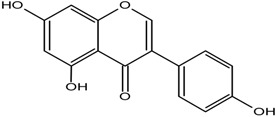
3	Daidzein	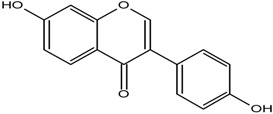
4	Glycitein	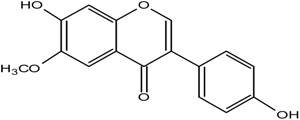
5	Formononetin	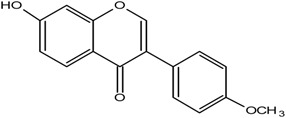

## Supplementary Materials

The following are available online.
