# Supplementary material for: Medicinal Potential of Isoflavonoids: Polyphenols That May Cure Diabetes
Source: Molecules. 2020 Nov 24;25(23):5491. doi: 10.3390/molecules25235491 (PMC7727648; doi:10.3390/molecules25235491)
Supplement: Supplementary file 1 [file molecules-25-05491-s001.pdf]

*Appendix A1*

# Medicinal Potential of Isoflavonoids: Polyphenols That May Cure Diabetes

**Qamar Uddin Ahmed [1,2,*], Abdul Hasib Mohd Ali [1], Sayeed Mukhtar [3,*], Meshari A. Alsharif [3], Humaira Parveen [3], Awis Sukarni Mohmad Sabere [1,2],** Mohamed Sufian Mohd. Nawi [1,2], **Alfi Khatib [1,2], Mohammad Jamshed Siddiqui [1,2], Abdulrashid Umar [4] and Alhassan Muhammad Alhassan [4]**

1 Department of Pharmaceutical Chemistry, Kulliyyah of Pharmacy, International Islamic University Malaysia, 25200 Kuantan, Pahang DM, Malaysia; quahmed@iium.edu.my (Q.U.A.); hasib76ali@gmail.com (A.H.M.A); awissabere@iium.edu.my (A.S.M.S.); msufian@iium.edu.my (M.S.M.N.); alfikhatib@iium.edu.my (A.K.); jamshed_siddiqui@iium.edu.my (M.J.S.)

2 Pharmacognosy Research Group, Department of Pharmaceutical Chemistry, Kulliyyah of Pharmacy, International Islamic University Malaysia, 25200 Kuantan, Pahang DM, Malaysia

3 Department of Chemistry, Faculty of Science, University of Tabuk, Tabuk 71491, Saudi Arabia; sayeed_mukhtar@hotmail.com (S.M.); me_alsharif@ut.edu.sa (M.A.A.); h.nabi@ut.edu.sa (H.P.)

4 Department of Pharmaceutical and Medicinal Chemistry, Faculty of Pharmaceutical Sciences, Usmanu Danfodiyo University, P M B: 2436 Sokoto, Nigeria; abdulrashid.umar@udusok.edu.ng (A.U.); alhasanmaudus@gmail.com (A.M.A.)

* Correspondence: authors emails: quahmed@iium.edu.my (Q.U.A.); sayeed_mukhtar@hotmail.com (S.M.)

| **Biological activity** | **Study** | **Cell Line/Animal (Reference)** |
|---|---|---|
| **1) Biochanin A** 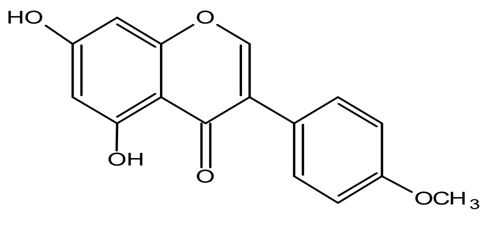  | *In vitro* | 1) Activation of PPAR alpha and gamma receptors (Wang et al., 2014)<br><br>2) Beneficial effect on insulin, hemoglobin and albumin glycosylation (Asgary et al., 2002)<br><br>3) Significantly potent inhibition on yeast $\alpha$-glucosidase in a dose dependent manner (Choi et al., 2010)<br><br>4) Activates PPARalpha, PPARgamma, and adipocyte differentiation (Shen et al., 2006)<br><br>5) Induces adipocyte differentiation through PPAR-gamma (Song et al., 2012)<br><br>6) Inhibits protein glycosylation in diabetic retinopathy (Asgary et al., 2002)<br><br>7) Insulin-resistant (IR) HepG2 cell model was used to evaluate the anti-hyperglycemic effects (Li et al., 2015) |

| | | |
|---|---|---|
| | | 8) Activates PPAR alpha and gamma and modulates adipocyte differentiation (Svjetlana et al., 2010) |
| | ***In vivo*** | 1) Induces insulin secretion (Lu et al., 2004; Azizi et al., 2014)<br><br>2) Antihyperglycemic effect on streptozotocin-diabetic rats (Harini et al, 2012)<br><br>3) Shows hypoglycemic and antilipemic activities and increases visfatin expression (Azizi et al., 2014)<br><br>4) Ameliorates dyslipidemia in streptozotocin-induced diabetic C57BL/6 mice by activating hepatic PPAR$\alpha$ (Qiu et al., 2012) |
| **2) Genistein**<br>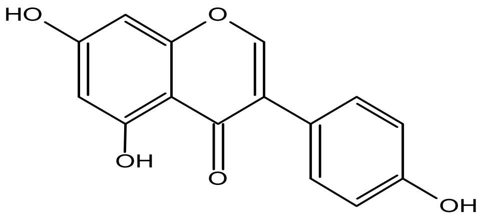 | ***In vitro*** | 1) Activates PPARalpha, PPARgamma, and adipocyte differentiation (Shen et al., 2006)<br><br>2)Insulin-resistant (IR) HepG2 cell model was used to evaluate the anti-hyperglycemic effects (Li et al., 2015)<br><br>3) Activates PPAR alpha and gamma and modulates adipocyte differentiation (Svjetlana et al., 2010)<br><br>4) Induces glucose uptake and accumulation in HL-60 and U-937 leukemic cell lines (Salas et al., 2013)<br><br>5) Inhibits insulin-stimulated glucose transport and decreases immunocytochemical labeling of GLUT4 carboxyl-terminus without affecting translocation of GLUT4 in isolated rat adipocytes (Smith et al., 1993)<br><br>6) Inhibits GLUT4-mediated glucose uptake in 3T3-L1 adipocytes (Bazuine et al., 2005)<br><br>7) Inhibitory mechanisms of flavonoids on insulin-stimulated glucose uptake in MC3T3-G2/PA6 adipose cells (Nomura et al., 2008)<br><br>8) Stimulates glucose-uptake in L6 myotubes (Lee et al., 2009)<br><br>9) Stimulates insulin secretion in normal mouse islets (Jonas et al., 1995) |

| | | |
|---|---|---|
| | | 10) Effects on the regulation of insulin-mediated glucose homeostasis in adipose tissue (differentiated 3T3-L1 adipocytes) (Wang et al., 2013)<br><br>11) Increases rapid glucose-stimulated insulin secretion in both insulin-secreting cell lines (INS-1 and MIN6) and mouse pancreatic islets (Liu et al., 2006)<br><br>12) Augments cyclic adenosine 3'5'-monophosphate(cAMP) accumulation and insulin release in MIN6 cells (Ohno et al., 1993)<br><br>13) Improves insulin secretory function of pancreatic beta-cells (clonal insulin secreting (INS-1E) cells) (Fu & Liu, 2009)<br><br>14) Inhibits tyrosine kinase in INS-1 cells, an insulin-secreting cell line (Neye & Verspohl, 1998)<br><br>15) Inhibits alpha-glucosidase (Lee & Lee, 2001)<br><br>16) Rapidly and temporarily induces SHARP-2 mRNA levels in a dose-dependent manner in rat H4IIE hepatoma cells (Haneishi et al., 2011)<br><br>17) Insulin-stimulated autophosphorylation of partially purified human insulin receptors from NIH 3T3/HIR 3.5 cells (Abler et al., 1992)<br><br>18) Inhibits inflammation and ameliorates endothelial dysfunction implicated in insulin resistance (Gao et al., 2013)<br><br>19) Antidiabetic and hypolipidemic effects through the PPAR pathways in obese Zucker rats and murine RAW 264.7 cells (Mezei et al., 2003)<br><br>20) Restricts leptin secretion from rat adipocytes (Szkudelski et al., 2005)<br><br>21) Counteracts the antilipolytic action of insulin in isolated rat adipocytes (Szkudelska et al., 2008)<br><br>22) Induces adipogenesis but inhibits leptin induction in human synovial fibroblasts (Relic et al., 2009)<br><br>23) Influence adenosine triphosphate levels in freshly isolated rat adipocytes (Szkudelska et al., 2011) |

| | | |
|---|---|---|
| | | 24) Effects of genistein on cytokine-induced pancreatic beta-cell damage. Treatment of RINm5F (RIN) rat insulinoma cells (Kim et al., 2007)<br><br>25) Augmentes insulin secretion in INS-1 cells (Lee et al., 2009)<br><br>26) Exhibits significant glucose consumption-enhancing effects in IR-HepG2 cells (Li et al., 2015)<br><br>27) Stimulates glucagon-like peptide-1 secretion in enteroendocrine NCI-H716 cells (Kwon et al., 2011)<br><br>28) Exerts antioxidantive and antidiabetic actions (Vedavanam et al., 1999)<br><br>29) Improves basal glucose uptake in HepG2 cells (Chen et al., 2010)<br><br>30) Suppresses LPS-induced inflammatory response through inhibiting NF-κB following AMP kinase activation in RAW 264.7 macrophages (Ji et al., 2012)<br><br>31) Inhibits islet tyrosine kinase activities and glucose-, 4alpha ketoisocaproic acid (KIC)- and sulphonylurea-stimulated insulin release (Persaud et al., 1999)<br><br>32) Enhance the insulin-stimulated sulfate uptake in articular chondrocytes (Claassen et al., 2008)<br><br>33) Shows antidiabetic properties (Getek et al., 2014)<br><br>34) Inhibits $\alpha$-glucosidase in a dose dependent manner (Choi et al., 2010) |
| | ***In vivo*** | 1) Induces endurance capacity and glucose utilization in streptozotocin-nicotinamide induced type 2 diabetic rat model (Bhattamisra et al., 2013)<br><br>2 Prevents T2D via a direct protective action on β-cells without alteration of periphery insulin sensitivity (Fu et al., 2012)<br><br>3) Impairs glucose tolerance and attenuates insulin sensitivity in normal mice (Wang et al., 2013) |

| | | |
|---|---|---|
| | | 4) Elevates insulin level and alters hepatic gluconeogenic and lipogenic enzyme activities in non-obese diabetic (NOD) mice (Choi et al., 2008)<br><br>5) Increases rapid glucose-stimulated insulin secretion (GSIS) in mouse pancreatic islets (Liu et al., 2006)<br><br>6) Induces pancreatic beta-cell proliferation through activation of multiple signalling pathways and prevents insulin-deficient diabetes in mice (Fu et al., 2010)<br><br>7) Effects on the insulin signalling pathway in the cerebral cortex of aged female rats (Moran et al., 2014)<br><br>8) Direct effects on β-cells (Gilbert & Liu, 2013)<br><br>9) Reduces blood glucose levels in streptozotocin-induced diabetic rats (Oh et al., 2010)<br><br>10) Shows effect on cardiovascular risk factors in postmenopausal women: relationship with the metabolic status (Villa et al., 2009)<br><br>11) Improves liver function and attenuates non-alcoholic fatty liver disease in a rat model of insulin resistance (Mohamed Salih et al., 2009)<br><br>12) Effects in a rat experimental model of postmenopausal metabolic syndrome (Bitto et al., 2009)<br><br>13) Shows an extensive therapeutical impact in the treatment of STZ-induced diabetic rats (Jesus et al., 2014)<br><br>14) Induces estrogen-like effects in ovariectomized (OVX) Sprague-Dawley rats (Al-Nakkash et al., 2010)<br><br>15) Antidiabetic and hypolipidemic effects through the PPAR pathways in obese Zucker rats and murine RAW 264.7 cells (Mezei et al., 2003)<br><br>16) Influences insulin secretion in ovariectomized rats (Nogowski et al., 2002)<br><br>17) Shows effect on insulin receptors in perfused liver of ovariectomized rats (Maćkowiak, Nogowski, & Nowak, 1999) |

| | | |
|---|---|---|
| | | 18) Shows hypoglycemic effect on postmenopausal women (Cheng et al., 2004)<br><br>19) Modulates hepatic glucose and lipid regulating enzyme activities in C57BL/KsJ-db/db mice (Ae Park et al., 2006)<br><br>20) Shows effects on blood glucose, antioxidant enzyme activities, and lipid profile in streptozotocin-induced diabetic rats (Lee, 2006)<br><br>21) Molecular effects of ER alpha- and beta-selective agonists on regulation of energy homeostasis in obese female Wistar rats (Weigt et al., 2013)<br><br>22) Suggested to improve insulin action in the skeletal muscle by targeting AMPK in a high fat-high fructose diet (HFFD)-fed mice model of insulin resistance (Arunkumar & Anuradha, 2012)<br><br>23) Reduces hyperglycemia and islet cell loss in a high-dosage manner in rats with alloxan-induced pancreatic damage (Yang et al., 2011)<br><br>24) Sensitizes hepatic insulin signalling and modulates lipid regulatory genes through p70 ribosomal S6 kinase-1 inhibition in high-fat-high-fructose diet-fed mice (Arunkumar et al., 2013)<br><br>25) Shows antihyperglycaemic and protective effects on streptozotocin-induced diabetic rats (Rauter et al., 2010)<br><br>26) Shows effect on insulin sensitivity and renal functional and structural injury in rats rendered insulin-resistant by feeding on a high-fructose diet for 60 days (Palanisamy et al., 2008) |
| **3) Daidzein**<br>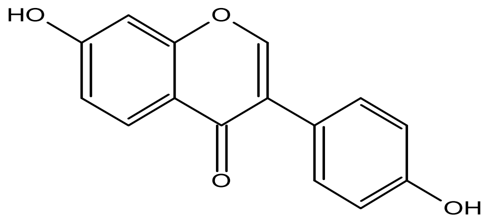 | *In vitro* | 1) Activates PPAR alpha and gamma and modulates adipocyte differentiation (Svjetlana et al., 2010)<br><br>2) Shows insulin-sensitizing action by activating peroxisome proliferator-activated receptor-$\gamma$ in 3T3-L1 adipocytes (Kwon et al., 2011)<br><br>3) Stimulates glucagon-like peptide-1 secretion in enteroendocrine NCI-H716 cells, thereby generates insulinotropic action (Kwon et al., 2011) |

| | | |
|---|---|---|
| | | 4) Regulates mechanisms of an insulin-inducible SHARP-2 gene by (S)-Equol, a metabolite of daidzein, induces rat SHARP-2 gene expression in H4IIE rat hepatoma cells (Haneishi et al., 2012)<br><br>5) Inhibits insulin- or insulin-like growth factor-1-mediated signalling in cell cycle progression of Swiss 3T3 cells (Higashi & Ogawara, 1994)<br><br>6) Enhances insulin-stimulated glucose uptake in 3T3-L1 adipocytes (Kwon et al., 2006)<br><br>7) PPAR transcriptional activity human promonocytic U-937 cells (Carrara et al., 2009)<br><br>8) Enhances the insulin-stimulated sulfate uptake in articular chondrocytes (Claassen et al., 2008)<br><br>9) Inhibits carbohydrate digestive enzymes *in vitro* (Park et al., 2013)<br><br>10) Exerts antidiabetic properties (Getek et al., 2014)<br><br>11) Inhibits yeast $\alpha$-glucosidase in a dose dependent manner (Choi et al., 2010)<br><br>12) Effects of daidzein and the daidzein metabolite, equol, on adipocyte differentiation and PPARgamma activation. In 3T3-L1 cells (Cho et al., 2010)<br><br>13) Promotes glucose uptake through glucose transporter 4 translocation to plasma membrane in L6 myocytes (Cheong et al., 2014a)<br><br>14) S-enantiomer Equol shows suppressive effects against alloxan-induced oxidative stress in INS-1 pancreatic β-cells (Horiuchi et al., 2014)<br><br>15) Exerts antidiabetic and hypolipidemic effects through the PPAR pathways in murine RAW 264.7 cells (Mezei et al., 2003)<br><br>16) A metabolite viz. 6,7,4'-trihydroxyisoflavone, suppresses adipogenesis in 3T3-L1 preadipocytes via ATP-competitive inhibition of PI3K (Seo et al., 2013)<br><br>17) Shows antihyperglycemic effect of equol, a daidzein derivative, in cultured L6 myocytes (Cheong et al., 2014b) |

| | | |
|---|---|---|
| | | 18) Soya phytochemical extract (Genistein and daidzein) inhibits intestinal glucose-uptake and acts as a preventive agent for glucose-induced lipid peroxidation (Vedavanam et al., 1999) |
| | *In vivo* | 1) Elevates insulin level and alters hepatic gluconeogenic and lipogenic enzyme activities in non-obese diabetic mice (Choi et al., 2008)<br><br>2) Modulates hepatic glucose and lipid regulating enzyme activities in C57BL/KsJ-db/db mice (Ae Park et al., 2006)<br><br>3) Reduces serum insulin and insulin resistance in obese diabetic rats (Bhathena & Velasquez, 2002)<br><br>4) Shows anti-obese and anti-diabetic effects on C57BL/6J mice fed with a high-fat diet (Zang et al., 2015)<br><br>5) Shows liver steatosis and expression of stearoyl-CoA desaturase 1 in diet-induced obesity in rats (Crespillo et al., 2011)<br><br>6) Alleviates postprandial hyperglycemia in streptozotocin-induced diabetic mice (Park et al., 2013)<br><br>7) Reduces blood glucose levels in streptozotocin-induced diabetic rats (Oh et al., 2010)<br><br>8) Improves glucose homeostasis in type 2 diabetic model mice (Cheong et al., 2014a)<br><br>9) Exerts antidiabetic and hypolipidemic effects through PPAR pathway in obese Zucker rats (Mezei et al., 2003)<br><br>10) Equol, a daidzein derivative, shows antihyperglycemic effect in obese-diabetic model ob/ob mice (Cheong et al., 2014b) |
| **4) Glycitein** | *In vitro* | 1) Shows PPAR transcriptional activity in human promonocytic U-937 cells (Carrara et al., 2009) |

| | | |
|---|---|---|
| 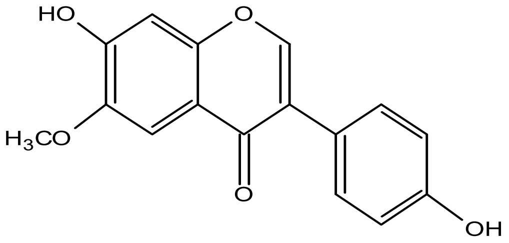 | | |
| | *In vivo* | 1) Anti-obese and anti-diabetic effects of a mixture of daidzin and glycitin on C57BL/6J mice fed with a high-fat diet (Zang et al., 2015)<br><br>2) Shows hypoglycaemic and hypolipidemic effects in streptozotocin-induced diabetic rats (Lee et al., 2000) |
| **5) Formononetin**<br><br>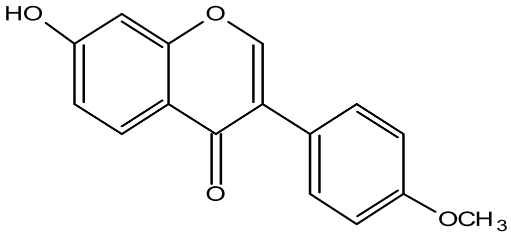 | *In vitro* | 1) Reduces caspase-3 levels in INS-1 cell line (Agyemang et al., 2013)<br><br>2) Insulin-resistant (IR) HepG2 cell model (Li et al., 2015)<br><br>3) Insulin-resistant (IR) HepG2 cell model was used to evaluate the anti-hyperglycaemic effects (Li et al., 2015)<br><br>4) Activates PPAR alpha and gamma and modulates adipocyte differentiation (Svjetlana et al., 2010)<br><br>5) Inhibits yeast $\alpha$-glucosidase in a dose dependent manner (Choi et al., 2010)<br><br>6) Chimeric and full-length PPAR constructs *in vitro* (Shen et al., 2006)<br><br>7) Mediates insulin-sensitizing effect through differentiation of 3T3-L1 preadipocyte and regulation of PPARgamma (Choi et al., 2013)<br><br>8) Improves insulin secretion by regulating pancreatic β-cell metabolism (Lee et al., 2019) |
| | *In vivo* | 1) Ameliorates dyslipidemia in streptozotocin-induced diabetic C57BL/6 mice by activating hepatic PPAR$\alpha$ (Qiu et al., 2012)<br><br>2) Potentiates the anti-hyperglycemic action of fangchinoline. Facilitates the fangchinoline-induced |

| | | |
|---|---|---|
| | | insulin releasein streptozotocin-diabetic mice (Ma et al., 2007) |

## References


- Abler, A.; Smith, J.A.; Randazzo, P.A.; Rothenberg, P.L.; Jarett, L. Genistein differentially inhibits postreceptor effects of insulin in rat adipocytes without inhibiting the insulin receptor kinase. *J. Biol. Chem.* **1992**, *267*, 3946–3951.
- Ae Park, S.; Choi, M.S.; Cho, S.Y.; Seo, J.S.; Jung, U.J.; Kim, M.J.; Sung, M.K.; Park, Y.B.; Lee, M.K. Genistein and daidzein modulate hepatic glucose and lipid regulating enzyme activities in C57BL/KsJ-db/db mice. *Life Sci.* **2006**, *79*, 1207–1213.
- Agyemang, K.; Han, L.; Liu, E.; Zhang, Y.; Wang, T.; Gao, X. Recent advances in *Astragalus membranaceus* antidiabetic research: Pharmacological effects of its phytochemical constituents. *Evid. Based Complement. Altern. Med.* **2013**, *2013*, 654643.
- Alhassan, M.A.; Ahmed, Q.U.; Latip, J.; Shah, S.A.A. A new sulphated flavone and other phytoconstituents from the leaves of *Tetracera indica* Merr, and their alpha-glucosidase inhibitory activity. *Nat. Prod. Res.* **2019**, *33*, 1–8.
- Al-Nakkash, L.; Markus, B.; Batia, L.; Prozialeck, W.C.; Broderick, T.L. Genistein induces estrogen-like effects in ovariectomized rats but fails to increase cardiac GLUT4 and oxidative stress. *J. Med. Food* **2010**, *13*, 1369–1375.
- Arunkumar, E.; Anuradha, C.V. Genistein promotes insulin action through adenosine monophosphate-activated protein kinase activation and p70 ribosomal protein S6 kinase 1 inhibition in the skeletal muscle of mice fed a high energy diet. *Nutr. Res.* **2012**, *32*, 617–625.
- Arunkumar, E.; Karthik, D.; Anuradha, C.V. Genistein sensitizes hepatic insulin signaling and modulates lipid regulatory genes through p70 ribosomal S6 kinase-1 inhibition in high-fat–high-fructose diet-fed mice. *Pharm. Biol.* **2013**, *51*, 815–824.
- Asgary, S.; Naderi, G.A.; Zadegan, N.S.; Vakili, R. The Inhibitory effects of pure flavonoids on *in vitro* protein glycosylation. *J. Herb. Pharm.* **2002**, *2*, 47–55.
- Ateba, S.B.; Mvondo, M.A.; Djiogue, S.; Zingué, S.; Krenn, L.; Njamen, D. A Pharmacological overview of alpinumisoflavone, a natural prenylated isoflavonoid. *Front. Pharm.* **2019**, *10*, 952.
- Azizi, R.; Goodarzi, M.T.; Salemi, Z. Effect of biochanin A on serum visfatin level of streptozocin-induced diabetic rats. *Iran. Red Crescent Med. J.* **2014**, 16, e15424.
- Bazuine, M.; van den Broek, P.J.; Maassen, J.A. Genistein directly inhibits GLUT4-mediated glucose uptake in 3T3-L1 adipocytes. *Biochem. Biophys. Res. Commun.* **2005**, *326*, 511–514.
- Bezek, S.; Ujhazy, E.; Mach, M.; Navarova, J.; Dubovicky, M. Developmental origin of chronic diseases: Toxicological implication. *Interdisc. Toxicol.* **2008**, *1*, 29–31.
- Bhathena, S.J.; Velasquez, M.T. Beneficial role of dietary phytoestrogens in obesity and diabetes. *Am. J. Clin. Nutr.* **2002**, *76*, 1191–1201.
- Bhattamisra, S.K.; Mohapatra, L.; Panda, B.P.; Parida, S. Effect of isoflavone rich soya seed extract on glucose utilization and endurance capacity in diabetic rat. *Diabetol. Croat.* **2013**, *42*, 42–52.
- Bitto, A.; Altavilla, D.; Bonaiuto, A.; Polito, F.; Minutoli, L.; Di Stefano, V.; Giuliani, D.; Guarini, S.; Arcoraci, V.; Squadrito, F. Effects of aglycone genistein in a rat experimental model of postmenopausal metabolic syndrome. *J. Endocrinol.* **2009**, *200*, 367–376.
- Carrara, V.S.; Amato, A.A.; Neves, F.A.; Bazotte, R.B.; Mandarino, J.M.; Nakamura, C.V.; Filho, B.P.; Cortez, D.A. Effects of a methanolic fraction of soybean seeds on the transcriptional activity of peroxisome proliferator-activated receptors (PPAR). *Braz. J. Med. Biol. Res.* **2009**, *42*, 545–550.
- Cederroth, C.R.; Nef, S. Soy, phytoestrogens and metabolism: A review. *Mol. Cell. Endocrinol.* **2009**, *304*, 30–42.
- Chen, Q.C.; Zhang, W.Y.; Jin, W.; Lee, I.S.; Min, B.S., Jung, H.J.; Na, M.; Lee, S.; Bae, K. Flavonoids and isoflavonoids from *Sophorae flos* improve glucose uptake *in vitro*. *Planta Med.* **2010**, *76*, 79–81.

- Cheng, S.Y.; Shaw, N.S.; Tsai, K.S.; Chen, C.Y. The hypoglycemic effects of soy isoflavones on postmenopausal women. *J. Womens Health* **2004**, *13*, 1080–1086.
- Cheong, S.H.; Furuhashi, K.; Ito, K.; Nagaoka, M.; Yonezawa, T.; Miura, Y.; Yagasaki, K. Antihyperglycemic effect of equol, a daidzein derivative, in cultured L6 myocytes and *ob*/*ob* mice. *Mol. Nutr. Food Res.* **2014**, 58, 267–277.
- Cheong, S.H.; Furuhashi, K.; Ito, K.; Nagaoka, M.; Yonezawa, T.; Miura, Y.; Yagasaki, K. Daidzein promotes glucose uptake through glucose transporter 4 translocation to plasma membrane in L6 myocytes and improves glucose homeostasis in Type 2 diabetic model mice. *J. Nutr. Biochem.* **2014**, 25, 136–143.
- Cho, K.W.; Lee, O.H.; Banz, W.J.; Moustaid-Moussa, N.; Shay, N.F.; Kim, Y.C. Daidzein and the daidzein metabolite, equol, enhance adipocyte differentiation and PPARgamma transcriptional activity. *J. Nutr. Biochem*. **2010**, *21*, 841–847.
- Choi, C.W.; Choi, Y.H.; Cha, M.R.; Yoo, D.S.; Kim, Y.S.; Yon, G.H.; Hong, K.S.; Kim, Y.H.; Ryu, S.Y. Yeast $\alpha$-glucosidase inhibition by isoflavones from plants of Leguminosae as an *in vitro* alternative to acarbose. *J. Agric. Food Chem*. **2010**, *58*, 9988–9993.
- Choi, E.J.; Kim, G.H. Antiproliferative activity of daidzein and genistein may be related to ER$\alpha$/c-erbB-2 expression in human breast cancer cells. *Mol. Med. Rep.* **2013**, *7*, 781–784.
- Choi, M.S.; Jung, U.J.; Yeo, J.; Kim, M.J.; Lee, M.K. Genistein and daidzein prevent diabetes onset by elevating insulin level and altering hepatic gluconeogenic and lipogenic enzyme activities in non-obese diabetic (NOD) mice. *Diabetes Metab. Res. Rev*. **2008**, *24*, 74–81.
- Choi, Y.; Noh, J.; Yun, S.W.; Kwon, Y.I.; Kim, Y.C. Effect of isoflavones from *Astragalus membranaceus* on 3T3-L1 adipocyte differentiation and insulin sensitivity. *FASEB J*. **2013**, *27*, 637–628.
- Chun, O.K.; Chung, S.J.; Song, W.O. Estimated dietary flavonoid intake and major food sources of U.S. adults. *J. Nutr.* **2007**, *137*, 1244–1252.
- Day, A.J.; Dupont, M.S.; Rhodes, M.J.C.; Morgan, M.R.A.; Williamson, G.; Ridley, S.; Rhodes, M. Deglycosylation of flavonoid and isoflavonoid glycosides by human small intestine and liver-glucosidase activity. *FEBS Lett.* **1998**, *436*, 71.
- Dinneen, S.; Gerich, J.; Rizza, R. Carbohydrate metabolism in non-insulin-dependent diabetes mellitus. *N. Engl. J. Med.* **1992**, *327*, 707–713.
- Dixon, R.A. Phytooestrogen. *Annu. Rev. Plant. Biol.* **2004**, *55*, 225–261.
- Dixon, R.A.; Summer, L.W. Legume natural products: Understanding and manipulating complex pathways for human and animal health. *Plant. Physiol*. **2003**, *131*, 878–885.
- Easom, R.A. CaM kinase II: A protein kinase with extraordinary talents germane to insulin exocytosis. *Diabetes* **1999**, *48*, 675–684.
- Fu, Z.; Gilbert, E.R.; Pfeiffer, L.; Zhang, Y.; Fu, Y.; Liu, D. Genistein ameliorates hyperglycemia in a mouse model of nongenetic type 2 diabetes. *Appl. Physiol. Nutr. Metab*. **2012**, *37*, 480–488.
- Fu, Z.; Liu, D. Long-term exposure to genistein improves insulin secretory function of pancreatic beta-cells. *Eur. J. Pharmacol*. **2009**, *616*, 321–327.
- Fu, Z.; Zhang, W.; Zhen, W.; Lum, H.; Nadler, J.; Bassaganya-Riera, J.; Jia, Z.; Wang, Y.; Misra, H.; Liu, D. Genistein induces pancreatic beta-cell proliferation through activation of multiple signaling pathways and prevents insulin-deficient diabetes in mice. *Endocrinology* **2010**, *151*, 3026–3037.
- Getek, M.; Czech, N.; Muc-Wierzgoń, M.; Grochowska-Niedworok, E.; Kokot, T.; Nowakowska-Zajdel, E. The active role of leguminous plant components in type 2 diabetes. *Evid. Based Complement. Alternat. Med*. **2014**, *2014*, 293961.
- Gilbert, E.R.; Liu, D. Anti-diabetic functions of soy isoflavone genistein: Mechanisms underlying its effects on pancreatic β-cell function. *Food Funct*. **2013**, *4*, 200–212.
- Harini, R.; Ezhumalai, M.; Pugalendi, K.V. Antihyperglycemic effect of biochanin A, a soy isoflavone, on streptozotocin-diabetic rats. *Eur. J. Pharmacol*. **2012**, *676*, 89–94.
- Hasan, M.M.; Ahmed, Q.U.; Soad, S.Z.M.; Tunna, T.S. Animal models and natural products to investigate *in vivo* and *in vitro* antidiabetic activity. *Biomed. Pharm.* **2018**, *101*, 833–841.
- Heikkinen, S.; Auwerx, J.; Argmann, C.A. PPARgamma in human and mouse physiology. *Biochim. Biophys. Acta* **2007**, 1771, 999–1013.

- Heinonen, S.; Wahala, K.; Adlercreutz, H. Identification of isoflavone metabolites dihydrodaidzein, dihydrogenistein, 6′-OH-O-dma, and cis-4-OH-equol in human urine by Gas Chromatography-Mass Spectroscopy using authentic reference compounds. *Anal. Biochem.* **1999**, *274*, 211–219.
- Higashi, K.; Ogawara, H. Daidzein inhibits insulin- or insulin-like growth factor-1-mediated signaling in cell cycle progression of Swiss 3T3 cells. *Biochim. Biophys. Acta* **1994**, *1221*, 29–35.
- Hoo, R.L.; Wong, J.Y.; Qiao, C.; Xu, A.; Xu, H.; Lam, K.S. The effective fraction isolated from *Radix astragali* alleviates glucose intolerance, insulin resistance and hypertriglyceridemia in db/db diabetic mice through its anti-inflammatory activity. *Nutr. Metab.* **2010**, *7*, 67.
- Horiuchi, H.; Harada, N.; Adachi, T.; Nakano, Y.; Inui, H.; Yamaji, R. S-equol enantioselectively activates cAMP-protein kinase A signaling and reduces alloxan-induced cell death in INS-1 pancreatic β-cells. *J. Nutr. Sci. Vitaminol.* **2014**, *60*, 291–296.
- Horn-Ross, P.L.; John, E.M.; Canchola, A.J.; Stewart, S.L.; Lee, M.M. Phytoestrogen intake and endometrial cancer risk. *J. Natl. Cancer Inst.* **2003**, *95*, 1158–1164.
- https://www.diabetesatlas.org/en/.
- https://www.who.int/news-room/fact-sheets/detail/diabetes.
- Hwang, C.S.; Kwak, H.S.; Lim, H.J.; Lee, S.H.; Kang, Y.S.; Choe, T.B.; Hur, H.G.; Han, K.O. Isoflavone metabolites and their in vitro dual functions: They can act as an estrogenic agonist or antagonist depending on the estrogen concentration. *J. Steroid Biochem. Mol. Biol.* **2006**, *101*, 246–253.
- Iwasaki, M.; Inoue, M.; Otani, T.; Sasazuki, S.; Kurashi, N.; Miura, T.; Yamoto, S.; Tsugane, S. Plasma isoflavone level and subsequent risk of breast cancer among Japanese women: A nested case-control study from Japan Public Health Cen-ter-base prospective study group. *J. Clin. Oncol.* **2008**, *26*, 1677–1683.
- Izumi, T.; Piskula, M.K.; Osawa, S.; Obata, A.; Tobe, K.; Saito, M.; Kataoka, S.; Kubota, Y.; Kikuchi, M. Soy isoflavone aglycones are absorbed faster and in higher amounts than their glucosides in humans. *J. Nutr.* **2000**, *130*, 1695–1699.
- Jang, C.H.; Lim, J.K.; Kim, J.H.; Park, C.S.; Kwon, D.Y.; Kim, Y.S.; Shin, D.H.; Kim, J.S. Change of Isoflavone Content during Manufacturing of Cheonggukjang, a Traditional Korean Fermented Soyfood. *Food Sci. Biotechnol.* **2006**, *15*, 643–646.
- Jesus, A.R.; Dias, C.; Matos, A.M.; de Almeida, R.F.; Viana, A.S.; Marcelo, F.; Ribeiro, R.T.; Macedo, M.P.; Airoldi, C.; Nicotra, F.; et al. Exploiting the therapeutic potential of 8-β-D-glucopyranosylgenistein: Synthesis, antidiabetic activity, and molecular interaction with islet amyloid polypeptide and amyloid β-peptide (1-42). *J. Med. Chem.* **2014**, *57*, 9463–9472.
- Jonas, J.C.; Plant, T.D.; Gilon, P.; Detimary, P.; Nenquin, M.; Henquin, J.C. Multiple effects and stimulation of insulin secretion by the tyrosine kinase inhibitor genistein in normal mouse islets. *Br. J. Pharm.* **1995**, *114*, 872–880.
- Kano, M.; Takayanagi, T.; Harada, K.; Sawada, S.; Ishikawa, F. Bioavailability of isoflavones after ingestion of soy beverages in healthy adults. *J. Nutr.* **2006**, *136*, 2291–2296.
- Kawakami, Y.; Tsurugasaki, W.; Nakamura, S.; Osada, K. Comparison of regulative functions between dietary soy isoflavones aglycone and glucoside on lipid metabolism in rats fed cholesterol. *J. Nutr. Biochem.* **2005**, *16*, 205–212.
- Kruk, I.; Aboul-Enein, H.Y.; Michalska, T.; Lichszteld, K.; Kładna, A. Scavenging of reactive oxygen species by the plant phenols genistein and oleuropein. *Luminescence* **2005**, *20*, 81–89.
- Kwon DY, Hong SM, Ahn IS, Kim MJ, Yang HJ, Park, S. Isoflavonoids and peptides from meju, long-term fermented soybeans, increase insulin sensitivity and exert insulinotropic effects *in vitro*. *Nutrition* **2011**, *27*, 244–252.
- Kwon, D.Y.; Jang, J.S.; Lee, J.E.; Kim, Y.S.; Shin, D.H.; Park, S. The isoflavonoid aglycone-rich fractions of Chungkookjang, fermented unsalted soybeans, enhance insulin signaling and peroxisome proliferator-activated receptor-gamma activity in vitro. *Biofactors* **2006**, *26*, 245–258.
- Lee, D.; Lee, D.H.; Choi, S.; Lee, J.S.; Jang, D.K.; Kang, K.S. Identification and isolation of active compounds from *Astragalus membranaceus* that improve insulin secretion by regulating pancreatic β-cell metabolism. *Biomolecules* **2019**, *9*, 618.
- Lee, D.S.; Lee, S.H. Genistein, a soy isoflavone, is a potent alpha-glucosidase inhibitor. *FEBS Lett.* **2001**, *501*, 84–86.

- Lee, H.S. Rat lens aldose reductase inhibitory activities of *Coptis japonica* root-derived isoquinoline alkaloids. *J. Agric. Food Chem.* **2002**, *50*, 7013–7016.
- Lee, J.S. Effects of soy protein and genistein on blood glucose, antioxidant enzyme activities, and lipid profile in streptozotocin-induced diabetic rats. *Life Sci.* **2006**, *79*, 1578–1584.
- Lee, M.S.; Kim, C.H.; Hoang, D.M.; Kim, B.Y.; Sohn, C.B.; Kim, M.R.; Ahn, J.S. Genistein-derivatives from *Tetracera scandens* stimulate glucose-uptake in L6 myotubes. *Biol. Pharm. Bull.* **2009**, *32*, 504–508.
- Lee, S.A.; Wen, W.; Xiang, Y.B.; Barnes, S.; Liu, D.; Cai, Q.; Zheng, W.; Xiao, O.S. Assessment of dietary isoflavone intake among middle-aged Chinese men. *J. Nutr.* **2007**, *137*, 1011–1016.
- Li, P.; Shi, X.; Wei, Y.; Qin, L.; Sun, W.; Xu, G.; Xu, T.; Liu, T. Synthesis and biological activity of isoflavone derivatives from chickpea as potent anti-diabetic agents. *Molecules* **2015**, *20*, 17016–17040.
- Liu, D.; Zhen, W.; Yang, Z.; Carter, J.D.; Si, H.; Reynolds, KA. Genistein acutely stimulates insulin secretion in pancreatic beta-cells through a cAMP-dependent protein kinase pathway. *Diabetes* **2006,** *55*, 1043–1050.
- Loureiro, G.; Martel, F. The effect of dietary polyphenols on intestinal absorption of glucose and fructose: Relation with obesity and type 2 diabetes. *J. Food Rev. Int.* **2019**, *35*, 390–406.
- Ma, W.; Nomura, M.; Nishioka, T.T.; Kobayashi, S. Combined effects of fangchinoline from *Stephania tetrandra* Radix and formononetin and calycosin from *Astragalus membranaceus* Radix on hyperglycemia and hypoinsulinemia in streptozotocin-diabetic mice. *Biol. Pharm. Bull.* **2007**, *30*, 2079–2083.
- Mackowiak, P.; Nogowski, L.; Nowak, K.W. Effect of isoflavone genistein on insulin receptors in perfused liver of ovariectomized rats. *J. Recept. Signal. Transduct. Res.* **1999**, *19*, 283–292.
- Matsuda, H.; Morikawa, T.; Yoshikawa, M. Antidiabetogenic constituents from several natural medicines. *Pure Appl. Chem.* **2002**, *74*, 1301–1308.
- Matveyenko, A.V.; Butler, P.C. Relationship between β-cell mass and diabetes onset. *Diabetes Obes. Metab.* **2008**, *10*, 23–31.
- Mayfield, J. Diagnosis and classification of diabetes mellitus: New criteria. *Am. Fam. Physician*. **1998**, *58*, 1355–1362.
- Mazur, W.; Adlercreutz, H. Overview of naturally occurring endocrine-active substances in the human diet in relation to human health. *Nutrition* **2000**, *16*, 654–658.
- Mezei, O.; Banz, W.J.; Steger, R.W.; Peluso, M.R.; Winters, T.A.; Shay, N. Soy isoflavones exert antidiabetic and hypolipidemic effects through the PPAR pathways in obese Zucker rats and murine RAW 264.7 cells. *J. Nutr.* **2003**, *133*, 1238–1243.
- Miadoková, E. Isoflavonoids—an overview of their biological activities and potential health benefits. *Interdiscip. Toxicol.* **2009**, *2*, 211–218.
- Molteni, A.; Brizio-Molteni, L.; Persky, V. *In vitro* hormonal effects of soybean isoflavones. *J. Nutr*. **1995**, 125, 751S–756S.
- Moran, J.; Garrido, P.; Cabello, E.; Alonso, A.; González, C. Effects of estradiol and genistein on the insulin signaling pathway in the cerebral cortex of aged female rats. *Exp. Gerontol*. **2014**, *58*, 104–112.
- Mulligan, A.A.; Welch, A.A.; McTaggart, A.A.; Bhaniani, A.; Bingham, S.A. Intakes and sources of soya foods and isoflavones in a UK population cohort study (EPIC-Norfolk). *Eur. J. Clin. Nutr.* **2007**, *61*, 248–254.
- Na, H.K.; Surh, Y.J. Peroxisome proliferator-activated receptor gamma (PPARgamma) ligands as bifunctional regulators of cell proliferation. *Biochem. Pharm.* **2003**, *66*, 1381–1391.
- Neye, H.; Verspohl, E.J. The specificity of tyrosine kinase inhibitors: Their effect on insulin release (short-term effect) and insulin mRNA (long-term effect) in an insulin-secreting cell line (INS-1). *Exp. Clin. Endocrinol. Diabetes*. **1998**, *106*, 292–298.
- Nogowski, L.; Nowak, K.W.; Kaczmarek, P.; Maćkowiak, P. The influence of coumestrol, zearalenone, and genistein administration on insulin receptors and insulin secretion in ovariectomized rats. *J. Recept. Signal. Transduct. Res.* **2002**, *22*, 449–457.
- Nomura, M.; Takahashi, T.; Nagata, N.; Tsutsumi, K.; Kobayashi, S.; Akiba, T.; Yokogawa, K.; Moritani, S.; Miyamoto, K. Inhibitory mechanisms of flavonoids on insulin-stimulated glucose uptake in MC3T3-G2/PA6 adipose cells. *Biol. Pharm. Bull.* **2008**, *31*, 1403–1409.

- Oh, T.W.; Kim, Y.A.; Jang, W.J.; Byeon, J.I.; Ryu, C.H.; Kim, J.O.; Ha, Y.L. Semipurified fractions from the submerged-culture broth of *Agaricus blazei* Murill reduce blood glucose levels in streptozotocin-induced diabetic rats. *J. Agric. Food Chem.* **2010**, *58*, 4113–4119.
- Ohno, T.; Kato, N.; Ishii, C.; Shimizu, M.; Ito, Y.; Tomono, S.; Kawazu, S. Genistein augments cyclic adenosine 3'5'-monophosphate (cAMP) accumulation and insulin release in MIN6 cells. *Endocr. Res.* **1993**, *19*, 273–285.
- Ørgaard, A.; Jensen, L. The effects of soy isofl avones on obesity. *Exp. Biol. Med.* **2008**, *233*, 1066–1080.
- Palanisamy, N.; Viswanathan, P.; Anuradha, C.V. Effect of genistein, a soy isoflavone, on whole body insulin sensitivity and renal damage induced by a high-fructose diet. *Ren. Fail.* **2008**, *30*, 645–654.
- Park, M.H.; Ju, J.W.; Park, M.J.; Han, J.S. Daidzein inhibits carbohydrate digestive enzymes *in vitro* and alleviates postprandial hyperglycemia in diabetic mice. *Eur. J. Pharm.* **2013**, *712*, 48–52.
- Persaud, S.J.; Harris, T.E.; Burns, C.J.; Jones, P.M. Tyrosine kinases play a permissive role in glucose-induced insulin secretion from adult rat islets. *J. Mol. Endocrinol.* **1999**, *22*, 19–28.
- Promden, W.; Monthakantirat, O.; Umehara, K.; Noguchi, H.; De-Eknamkul, W. Structure and antioxidant activity relationships of isoflavonoids from *Dalbergia parviflora*. *Molecules* **2014**, *19*, 2226–2237.
- Qiu, L.; Ye, H.; Chen, L.; Hong, Y.; Zhong, F.; Zhang, T. Red clover extract ameliorates dyslipidemia in streptozotocin-induced diabetic C57BL/6 mice by activating hepatic PPAR$\alpha$. *Phytother. Res.* **2012**, *26*, 860–864.
- Rauter, A.P.; Martins, A.; Borges, C.; Mota-Filipe, H.; Pinto, R.; Sepodes, B.; Justino, J. Antihyperglycaemic and protective effects of flavonoids on streptozotocin-induced diabetic rats. *Phytother. Res.* **2010**, 24, S133–S138.
- Reiter, E.; Beck, V.; Medjakovic, S.; Mueller, M.; Jungbauer, A. Comparison of hormonal activity of isoflavone-containing supplements used to treat menopausal complaints. *Menopause* **2009**, *16*, 1049–1060.
- Relic, B.; Zeddou, M.; Desoroux, A.; Beguin, Y.; de Seny, D.; Malaise, M.G. Genistein induces adipogenesis but inhibits leptin induction in human synovial fibroblasts. *Lab. Invest.* **2009**, *89*, 811–822.
- Rochfort, S.; Panozzo, J. Phytochemicals for health, the role of pulses. *J. Agric. Food Chem.* **2007**, *55*, 7381–7994.
- Sabudak, T.; Guler, N.; Trifolium, L. A review on its phytochemical and pharmacological profile. *Phytother. Res.* **2009**, *23*, 439–446.
- Salih, M.; Nallasamy, P.; Muniyandi, P.; Periyasami, V.; Venkatraman, A.C. Genistein improves liver function and attenuates non-alcoholic fatty liver disease in a rat model of insulin resistance. *J. Diabetes*. **2009**, *1*, 278–287.
- Santaguida, P.L.; Balion, C.; Hunt, D. Diagnosis, prognosis, and treatment of impaired glucose tolerance and impaired fasting glucose. *Evid. Rep. Technol. Assess.* **2008**, *12*, 1–11.
- Seo, S.G.; Yang, H.; Shin, S.H.; Min, S.; Kim, Y.A.; Yu, J.G.; Lee, D.E.; Chung, M.; Heo, Y.; Kwon, J.Y.; et al. A metabolite of daidzein, 6,7,4'-trihydroxyisoflavone, suppresses adipogenesis in 3T3-L1 preadipocytes via ATP-competitive inhibition of PI3K. Mol. *Nutr. Food Res.* **2013**, *57*, 1446–1455.
- Setchell, K.D.R.; Brown, N.M.; Zimmer-Nechemias, L.; Brashear, W.T.; Wolfe, B.E.; Kirschner, A.S.; Heubi, J.E. Evidence for lack of absorption of soy isoflavone glycosides in humans, supporting the crucial role of intestinal metabolism for bioavailability. *Am. J. Clin. Nutr.* **2002**, *76*, 447–453.
- Shen, P.; Liu, M.H.; Ng, T.Y.; Chan, Y.H.; Yong, E.L. Differential effects of isoflavones, from *Astragalus membranaceus* and *Pueraria thomsonii*, on the activation of PPARalpha, PPARgamma, and adipocyte differentiation *in vitro*. *J. Nutr.* **2006**, *136*, 899–905.
- Smith, R.M.; Tiesinga, J.J.; Shah, N.; Smith, J.A.; Jarett, L. Genistein inhibits insulin-stimulated glucose transport and decreases immunocytochemical labeling of GLUT4 carboxyl-terminus without affecting translocation of GLUT4 in isolated rat adipocytes: Additional evidence of GLUT4 activation by insulin. *Arch. Biochem. Biophys.* **1993**, *300*, 238–246.
- Song, M.K.; Roufogalis, B.D.; Huang, T.H.W. Modulation of diabetic retinopathy pathophysiology by natural medicines through PPAR-$\gamma$-related pharmacology. *Br. J. Pharm.* **2012**, *165*, 4–19.
- Spranger, J.; Kroke, A.; Möhlig, M.; Hoffmann, K.; Bergmann, M.M.; Ristow, M.; Boeing, H.; Pfeiffer, A.F.H. Inflammatory cytokines and the risk to develop type 2 diabetes: Results of the prospective population-based European Prospective Investigation into Cancer and Nutrition (EPIC)-Potsdam study. *Diabetes* **2003**, 52, 812–817.

- Sun, L.; Miao, M. Dietary polyphenols modulate starch digestion and glycaemic level: A review. *Crit. Rev. Food. Sci. Nutr.* **2020**, *60*, 541–555.
- Surh, J.; Kim, M.J.; Koh, E.; Kim, Y.K.L.; Kwon, H. Estimated intakes of isoflavones and coumestrol in Korean population. *Int. J. Food Sci. Nutr.* **2006**, *57*, 325–344.
- Svjetlana, M.; Monika, M.; Alois, J. Potential Health-modulating effects of isoflavones and metabolites via activation of PPAR and AhR. *Nutrients* **2010**, *2*, 241–279.
- Szkudelska, K.; Nogowski, L.; Szkudelski, T. Genistein, a plant-derived isoflavone, counteracts the antilipolytic action of insulin in isolated rat adipocytes. *J. Steroid Biochem. Mol. Biol.* **2008**, *109*, 108–114.
- Szkudelski, T.; Nogowski, L.; Pruszyńska-Oszmałek, E.; Kaczmarek, P.; Szkudelska, K. Genistein restricts leptin secretion from rat adipocytes. *J. Steroid Biochem. Mol. Biol.* **2005**, *96*, 301–307.
- Takata, Y.; Maskarinec, G.; Franke, A.; Nagata, C.; Shimizu, H. A comparison of dietary habits among women in Japan and Hawaii. *Public Health Nutr.* **2004**, *7*, 319–326.
- Teles, R.B.A.; Diniz, T.C.; Pinto, T.C.C.; Júnior, R.G.O.; Silva, M.G.; de Lavor, E.M.; Fernandes, A.W.C.; de Oliveira, A.P., Ribeiro, F.P.R.D.; da Silva, A.A.M.; et al. Flavonoids as therapeutic agents in alzheimer's and parkinson's diseases: A systematic review of preclinical evidences. *Oxid. Med. Cell. Longev.* **2018**, *2018*, 7043213.
- Tontonoz, P.; Spiegelman, B.M. Fat and beyond: The diverse biology of PPARγ. *Annu. Rev. Biochem.* **2008**, *77*, 289–312.
- Tsunoda, N.; Pomeroy, S.; Nestel, P. Absorption in humans of isoflavones from soy and red clover is similar. *J. Nutr.* **2002**, *132*, 2199.
- Vedavanam, K.; Srijayanta, S.; O'Reilly, J.; Raman, A.; Wiseman, H. Antioxidant action and potential antidiabetic properties of an isoflavonoid-containing soyabean phytochemical extract (SPE). *Phytother. Res.* **1999**, *13*, 601–608.
- Wang, M.; Gao, X.J.; Zhao, W.W.; Zhao, W.J.; Jiang, C.H.; Huang, F.; Kou, J.P.; Liu, B.L.; Liu, K. Opposite effects of genistein on the regulation of insulin-mediated glucose homeostasis in adipose tissue. *Br. J. Pharm.* **2013**, *170*, 328–340.
- Wang, Y.; Han, Y.; Teng, W.; Zhao, X.; Li, Y.; Wu, L.; Li, D.; Li, W. Expression quantitative trait loci infer the regulation of isoflavone accumulation in soybean (*Glycine max* L. Merr.) seed. *BMC Genom.* **2014**, *15*, 680.
- Wei, Y.; Li, P.; Li, B.; Gao, J.; Wang, D.; Qin, L.; Sun, W.; Xu, Y.; Shi, H.; Xu, T.; et al. Study of the hypoglycemic activity of derivatives of isoflavones from *Cicer arietinum* L. *Evid. Based Complement. Altern. Med.* **2017**, *2017*, 8746823.
- Weigt, C.; Hertrampf, T.; Kluxen, F.M.; Flenker, U.; Hülsemann, F.; Fritzemeier, K.H.; Diel, P. Molecular effects of ER alpha- and beta-selective agonists on regulation of energy homeostasis in obese female Wistar rats. *Mol. Cell. Endocrinol.* **2013**, *377*, 147–158.
- Yang, W.; Wang, S.; Li, L.; Liang, Z.; Wang, L. Genistein reduces hyperglycemia and islet cell loss in a high-dosage manner in rats with alloxan-induced pancreatic damage. *Pancreas* **2011**, *40*, 396–402.
- Zang, Y.; Igarashi, K.; Yu, C. Anti-obese and anti-diabetic effects of a mixture of daidzin and glycitin on C57BL/6J mice fed with a high-fat diet. *Biosci. Biotechnol. Biochem.* **2015**, *79*, 117–123.
- Zheng, V.; Lee, S.O.; Murphy, P.A.; Hendrich, S.; Verbruggen, M.A. The apparent absorptions of isoflavone glucosides and aglucons are similar in women and are increased by rapid gut transit time and low fecal isoflavone degradation. *J. Nutr.* **2004**, *134*, 2534.
- Zubik, L.; Meydani, M. Bioavailability of soybean isoflavones from aglycone and glucoside forms in American women. *Am. J. Clin. Nutr.* **2003**, *77*, 1459–1465.
